# Composition analysis of β-(In*_x_*Ga_1-*x*_)_2_O_3_ thin films coherently grown on (010) β-Ga_2_O_3_ via mist CVD

**DOI:** 10.1080/14686996.2024.2414733

**Published:** 2024-10-16

**Authors:** Hiroyuki Nishinaka, Yuki Kajita, Shoma Hosaka, Hiroki Miyake

**Affiliations:** a Faculty of Electrical Engineering and Electronics, Kyoto Institute of Technology, Kyoto, Japan; b Department of Electronics, Kyoto Institute of Technology, Kyoto, Japan; c Power Electronics R & D Div. 2, MIRISE Technologies Corporation, Aichi, Japan; d Kyoto Lab for a Greener Future, Kyoto Institute of Technology, Kyoto, Japan

**Keywords:** Ga_2_O_3_, In_2_O_3_, alloying, coherent growth, epitaxial growth, mist CVD

## Abstract

This study investigates the compositional analysis and growth of β-(In_*x*_Ga_1-*x*_)_2_O_3_ thin films on (010) β-Ga_2_O_3_ substrates using mist chemical vapor deposition (CVD), including the effects of the growth temperature. We investigated the correlation between In composition and *b*-axis length in coherently grown films, vital for developing high-electron-mobility transistors and other devices based on β-(In_*x*_Ga_1-*x*_)_2_O_3_. Analytical techniques, including X-ray diffraction (XRD), reciprocal space mapping, and atomic force microscopy, were employed to evaluate crystal structure, strain relaxation, and surface morphology. The study identified a linear relationship between In composition and *b*-axis length in coherently grown films, facilitating accurate composition determination from XRD peak positions. The films demonstrated high surface flatness with root-mean-square roughness below 0.6 nm, though minor relaxation and granular features emerged at higher In compositions (*x* = 0.083) at the growth temperature of 750°C. XRD results revealed that lattice relaxation were observed at a growth temperature of 700°C despite low In composition. In contrast, at 800°C, the In composition was higher than at 750°C, and coherent growth was achieved. The surface morphology was the flattest at 750°C. These findings indicate that the growth temperature plays a crucial role in the mist CVD growth of β-(In_*x*_Ga_1-*x*_)_2_O_3_ thin films. This study offers insights into the relationship between In composition and lattice parameters in coherently grown β-(In_*x*_Ga_1-*x*_)_2_O_3_ films, as well as the effect of growth conditions, contributing to the advancement of ultra-wide bandgap semiconductor device development.

## IMPACT STATEMENT

This paper presents innovative growth techniques for β-(InxGa1-x)₂O₃ thin films, enabling precise In composition control and enhancing potential applications in next-generation power-switching devices.

## Graphical abstract

**Figure:**
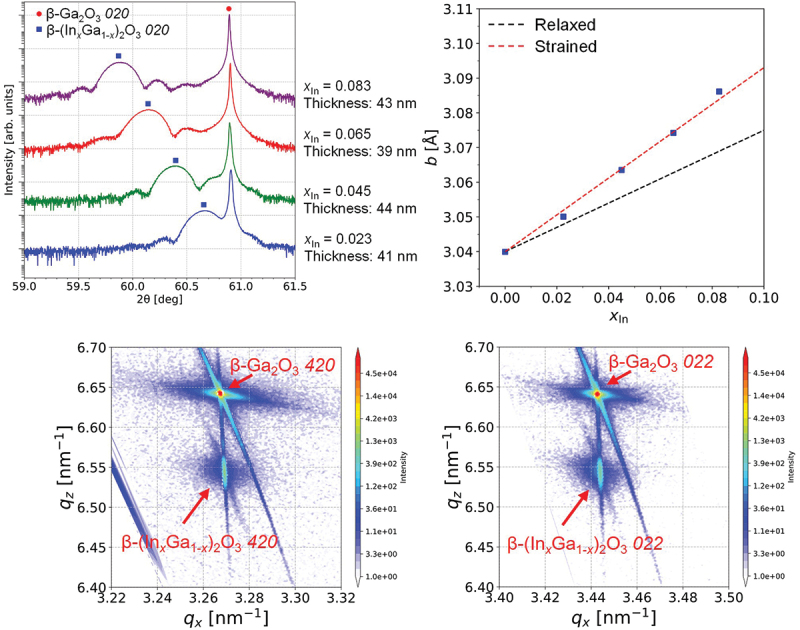

## 1. Introduction

Gallium oxide, an ultra-wide bandgap semiconductor with a bandgap ranging from 4.6 to 5.3 eV, has garnered considerable attention [1,2] due to its potential for high-efficiency power switching devices and ultraviolet photodetectors [3,4]. Among the five polymorphs of Ga_2_O_3_—α, β, γ, δ, and κ(ε) phases [5]—β-Ga_2_O_3_ stands out as the thermodynamically most stable phase and the only one capable of forming bulk single crystals via melt growth techniques [6–8]. When sliced and polished into wafers, these high-quality bulk crystals exhibit excellent performance across various applications [9–12]. These single-crystal bulks can be doped with various impurities to achieve a wide range of conductivity, from high carrier concentrations (10^18^ to 10^19^ cm^−3^) using Si and Sn as donors [13,14], to semi-insulating properties using compensating impurities such as Fe [15] and Mg [16]. This controllable conductivity is crucial for the operation of β-Ga_2_O_3_ devices.

Specifically focusing on the β polymorph, β-Ga_2_O_3_ has been successfully implemented in various power devices, including Schottky barrier diodes [9], fin-field-effect transistors (FET) [10], and modulation-doped FETs [11]. However, Ga_2_O_3_ lacks p-type conductivity, which poses a significant challenge. Heterojunction p-n devices using NiO as the p-type oxide have been developed to mitigate this issue [17,18]. However, high-electron-mobility transistors (HEMTs) using two-dimensional electron gas (2DEG), such as modulation-doped FETs for high-frequency applications, offer promising applications that do not require p-type Ga_2_O_3_. Developing β-Ga_2_O_3_ alloy is crucial for these HEMTs. Studies on incorporating Al_2_O_3_ into Ga_2_O_3_ have aimed to increase the bandgap [19,20], resulting in multiple device demonstrations [21–23]. The 2DEG density in HEMTs, which influences the on-resistance, depends on the free electron concentration in the modulation-doped layer and the conduction band offset between β-(Al_*x*_Ga_1-*x*_)_2_O_3_ and β-Ga_2_O_3_. Higher free electron concentrations and larger band offsets yield higher 2DEG densities. However, achieving larger band offsets necessitates higher Al concentrations, introducing challenges such as limited film thickness due to significant lattice mismatch and insufficient impurity activation at high Al compositions. Hence, β-(Al_*x*_Ga_1-*x*_)_2_O_3_/β-(In_*y*_Ga_1-*y*_)_2_O_3_ heterostructures have been proposed to overcome these challenges, combining alloys with In_2_O_3_ [24] to achieve larger band offsets while facilitating lower Al compositions. This approach potentially enhances free electron concentrations through enhanced carrier confinement.

Oshima et al. conducted an analysis of Al composition and its correlation with lattice parameters in β-(Al_*x*_Ga_1-*x*_)_2_O_3_. They established equations to calculate Al composition *x* from strained lattice parameters for coherently grown (010) β-(Al_*x*_Ga_1-*x*_)_2_O_3_ films [25]. This comprehensive approach, which included both composition analysis and verification of the composition-lattice parameter relationship, enables Al composition determination through readily accessible X-ray diffraction (XRD) techniques. Consequently, it allows indirect assessment of bandgap variations and estimation of band offsets, critical parameters for heterostructure design and optimization.

Extending this analytical approach to β-(In_*x*_Ga_1-*x*_)_2_O_3_ is crucial for advancing HEMT technology. Existing research highlights its potential despite fewer studies on β-(In_*x*_Ga_1-*x*_)_2_O_3_ than β-(Al_*x*_Ga_1-*x*_)_2_O_3_. Annoz et al. demonstrated coherent growth of β-(In_*x*_Ga_1-*x*_)_2_O_3_ on (100) β-Ga_2_O_3_ substrates using MOCVD, achieving coherent growth up to *x* = 0.035 [26]. Similarly, Mazzolini et al. reported coherent growth on (010) β-Ga_2_O_3_ substrates using MBE up to *x* = 0.1 (thickness: 122 ± 2 nm) [24]. While these studies presented relationships between In composition and lattice parameters, they lacked comprehensive verification or explicit equations for these relationships.

Our group has demonstrated various β-Ga_2_O_3_ growth techniques using mist CVD, including coherent growth of (010) β-(Al_*x*_Ga_1-*x*_)_2_O_3_ [27], rapid growth of β-Ga_2_O_3_ [28], and β-(Al_*x*_Ga_1-*x*_)_2_O_3_/β-(In_*y*_Ga_1-*y*_)_2_O_3_ coherently grown heterojunctions on (010) β-Ga_2_O_3_ substrates [29]. However, the growth and composition control of β-(In_*x*_Ga_1-*x*_)_2_O_3_ present unique challenges in mist CVD. In this technique, the final film composition does not exactly mirror the solution concentration due to vapor-phase reactions occurring via the Leidenfrost effect [30,31] and the differing reaction rates of each precursor. Therefore, a comprehensive analysis of alloy thin film compositions grown by mist CVD is critical.

In this study, we employed mist CVD to grow β-(In_*x*_Ga_1-*x*_)_2_O_3_ on (010) β-Ga_2_O_3_ substrates and investigated the effects of In composition and growth temperature on coherent growth. Specifically, we aim to elucidate the relationship between In composition and XRD peak positions under conditions of coherent growth for β-(In_*x*_Ga_1-*x*_)_2_O_3_, analogous to the approach established for β-(Al_*x*_Ga_1-*x*_)_2_O_3_. This understanding is essential for alloying Ga_2_O_3_ to tune the bandgap, crucial for optimizing device performance in applications such as HEMTs. By establishing equations relating In composition to lattice parameters in strained β-(In_*x*_Ga_1-*x*_)_2_O_3_ films, we seek to enable precise control of composition, bandgap, and band offsets in these heterostructures. This capability is essential for optimizing β-(In_*x*_Ga_1-*x*_)_2_O_3_-based HEMTs, potentially leading to devices with enhanced performance characteristics. Through this comprehensive approach, we address the unique challenges of β-(In_*x*_Ga_1-*x*_)_2_O_3_ growth in mist CVD while advancing the fundamental understanding necessary for future device applications.

## 2. Experimental

β-(In_*x*_Ga_1-*x*_)_2_O_3_ thin films were grown on (010) β-Ga_2_O_3_ substrates using a hot-wall type mist CVD. GaCl_3_ and InCl_3_ served as Ga and In precursors, respectively, dissolved in a de-ionized water solution to maintain a total concentration of 0.5 M, with varying Ga and In concentrations. The reaction zone comprised a quartz tube heated by a tubular furnace. The quartz tube had an inner diameter of 40 mm. Nitrogen (N_2_) gas at a flow rate of 10 L/min was used as the carrier gas. The growth was conducted at 750°C for 1 min. X-ray diffraction (XRD, Bruker D8Discover) 2θ-ω scans and reciprocal space mapping (RSM) were employed to investigate the crystalline structures of the thin films. Atomic force microscopy (AFM, SII Nanonavi E-Sweep) was used to observe surface morphologies. Moreover, Rutherford backscattering spectroscopy (RBS) estimated the In composition of the β-(In_*x*_Ga_1-*x*_)_2_O_3_ thin films.

## 3. Results and discussion

**Figure 1.**
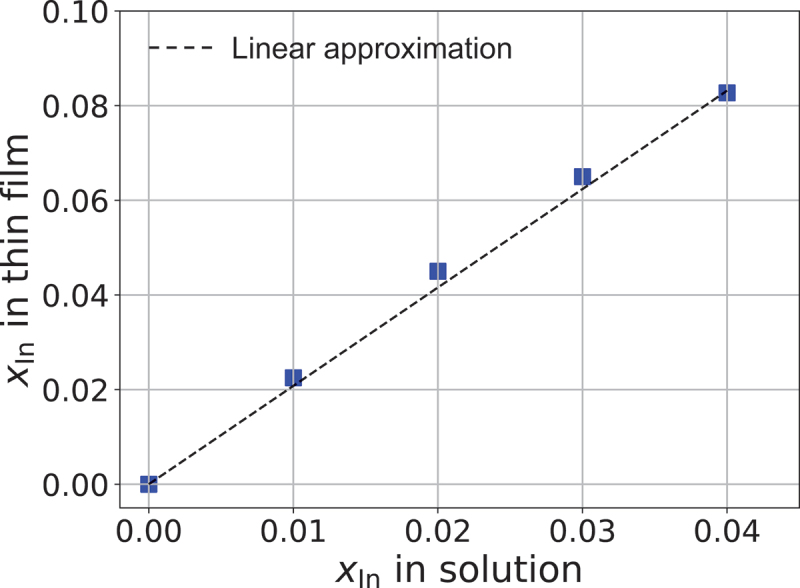
Relationship between In concentration in the solution and the in composition in the thin film. The dashed line represents the linear approximation.

We initially examined the impact of solution concentration on the compositions of β-(In_*x*_Ga_1-*x*_)_2_O_3_ thin films. In mist CVD, the composition of the thin film can be controlled by solution concentration. However, the thin film composition does not exactly mirror the solution concentration due to the vapor-phase reaction occurring via the Leidenfrost effect and the differing reaction rates of each precursor in the vapor phase. Therefore, analysing the compositions of alloy thin films grown by mist CVD is critical. Figure 1 illustrates the In composition of β-(In_*x*_Ga_1-*x*_)_2_O_3_ thin films, as measured by RBS, as a function of the In concentration in the solution. The In composition of the thin films increased linearly with the In concentration of the solution, although the In incorporation into the thin films exceeded the In concentration of the solution. This discrepancy likely arises from the vapor-phase reaction process and the different reaction rates of GaCl_3_ and InCl_3_ in the vapor phase.

**Figure 2.**
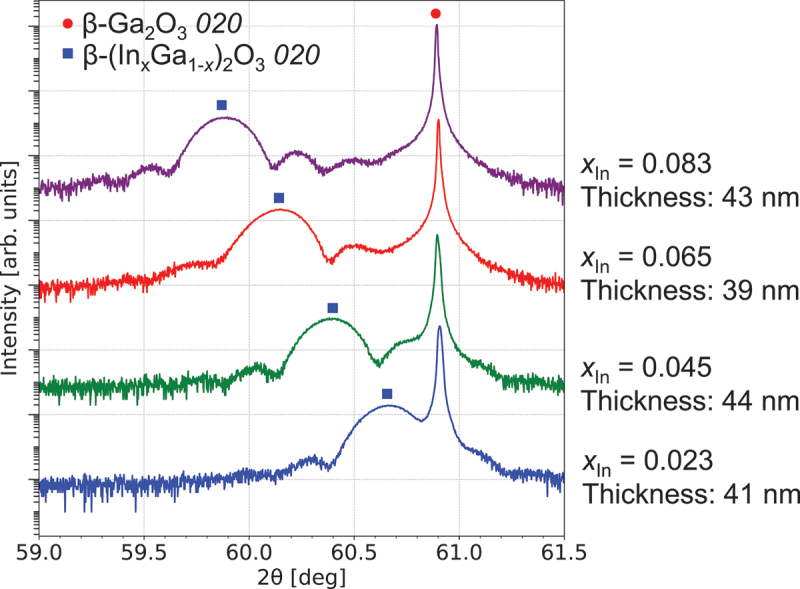
XRD 2θ-ω patterns for β-(In_*x*_Ga_1-*x*_)_2_O_3_ thin films with varying In compositions.

XRD 2θ-ω scans were performed to investigate diffraction peak positions, and RSM was conducted to confirm coherent growth. Figure 2 presents the XRD 2θ-ω scans of β-(In_*x*_Ga_1-*x*_)_2_O_3_ thin films. All diffraction patterns exhibited Laue oscillation, with diffraction peaks shifting to lower angle sides as the In composition increased. The film thickness, calculated from the Laue oscillations, ranged from 39 to 44 nm.

**Figure 3.**
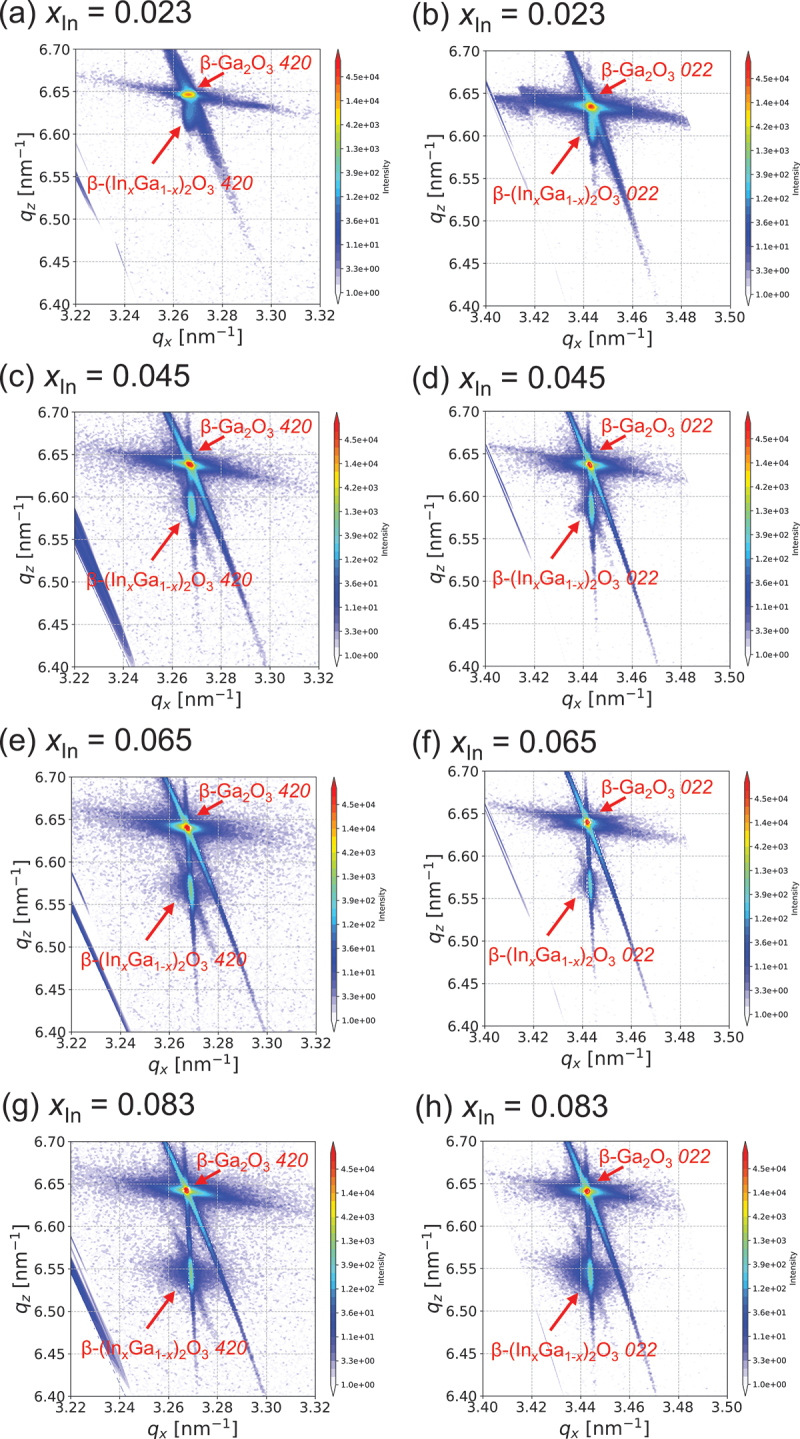
XRD RSM results of β-(In_*x*_Ga_1-*x*_)_2_O_3_ thin films with different In compositions, focusing on the *420* and *022* reflections.

Mazzolini et al. reported anisotropic strain relaxation in (010) β-(In_*x*_Ga_1-*x*_)_2_O_3_ thin films [24]. Specifically, their study demonstrated that strain relaxation begins at different film thicknesses for the *a*-axis and *c*-axis. To investigate this phenomenon in our samples and compare with their findings, we conducted reciprocal space mapping (RSM) measurements of our β-(In_*x*_Ga_1-*x*_)_2_O_3_ thin films. We measured around the (420) plane to analyse the in-plane *a*-axis and around the (022) plane for the in-plane *c*-axis. Figure 3 shows the XRD RSM results for the β-(In_*x*_Ga_1-*x*_)_2_O_3_ thin films. The diffraction spots of the β-(In_*x*_Ga_1-*x*_)_2_O_3_ thin films were positioned directly beneath the diffraction spots of the *420* and *022* for the substrate, indicating coherent growth for all compositions. The *022* spot exhibited slight initial relaxation at an In composition of *x* = 0.083, while the *420* spot appeared less relaxed. However, this difference was subtle and not sufficiently pronounced for a definitive evaluation. All films demonstrated predominantly coherent growth, making them suitable for compositional assessment based on XRD peak positions.

**Figure 4.**
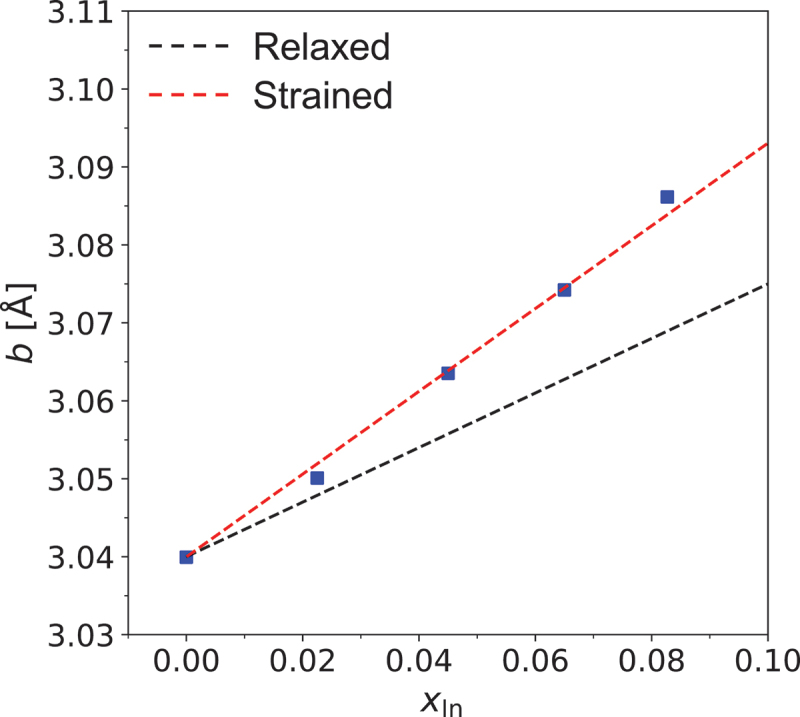
Variation of the *b*-axis lattice parameter as a function of in composition *x* in β-(In_*x*_Ga_1-*x*_)_2_O_3_ thin films coherently grown on (010)β-Ga_2_O_3_ substrates. The black dashed line represents the relaxed β-(In_*x*_Ga_1-*x*_)_2_O_3_ lattice parameter relationship based on the results of Kranert [32], while the red dashed line illustrates the calculated equation for the strained β-(In_*x*_Ga_1-*x*_)_2_O_3_.

Figure 4 presents a plot of the *b*-axis length as a function of In composition in the films. The red dashed line depicts the theoretical relationship between the *b*-axis and In composition for strained β-(In_*x*_Ga_1-*x*_)_2_O_3_ growth, while the black dashed line illustrates the relationship for relaxed growth conditions. The relaxed relationship derives from the findings of Kranert et al. for β-(In_*x*_Ga_1-*x*_)_2_O_3_ [32], with an adjustment of the reported *b*-axis length for Ga_2_O_3_ from 3.045 Å to 3.040 Å based on our XRD measurements of the substrate. The strained relationship line was developed using the results of Kranert et al. [32], employing the methodology previously used by Oshima et al. for β-(Al_*x*_Ga_1-*x*_)_2_O_3_ [25]. The resulting equation, $x\;≅\;-5.726+1.884\times b_{c}$(Å), relates x of β-(In_*x*_Ga_1-*x*_)_2_O_3_ thin films to the *b*-axis length $b_{c}\;$of coherent growth. As illustrated, the *b*-axis lengths and In compositions (*x*) exhibited a linear correlation that aligns closely with the strained relationship. This result demonstrates that the *b*-axis length of coherently grown β-(In_*x*_Ga_1-*x*_)_2_O_3_ can be used to determine In composition, thereby offering a valuable tool for characterizing and optimizing β-(In_*x*_Ga_1-*x*_)_2_O_3_ based devices and advancing future technological developments in this area.

A slight relaxation was initiated along the *c*-axis at *x* = 0.083, suggesting that the critical thickness for *x* = 0.083 was approximately 40 nm. However, Cao et al. have calculated that the critical thickness for *x* = 0.083 exceeded 400 nm [33]. Mazzolini et al. have reported coherent growth for In compositions of up to 10% at a film thickness of 120 nm when grown on (010) β-Ga_2_O_3_ by molecular beam epitaxy [24]. While their data showed coherent growth through spot positions, a slight broadening was evident. We suggest that this broadening indicates the onset of minor relaxation, although the authors did not explicitly address it. We considered any minimal broadening as a sign of relaxation, leading us to believe that the critical thickness might be lower than indicated by theoretical calculations. A thorough evaluation of critical thickness would require detailed studies involving various film thicknesses. Future research will explore these evaluations more comprehensively. Understanding these relaxation mechanisms and accurately determining critical thicknesses are essential for optimizing the growth of high-quality β-(In_*x*_Ga_1-*x*_)_2_O_3_ films for device applications.

**Figure 5.**
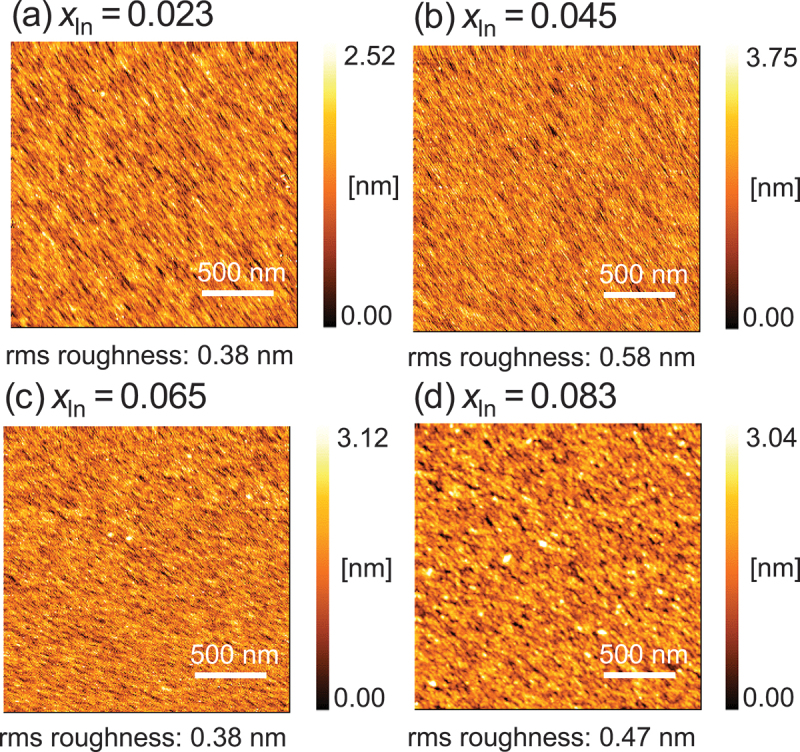
AFM images of β-(In_*x*_Ga_1-*x*_)_2_O_3_ thin films with varying In composition.

High surface flatness is essential for forming heterojunctions because rough interfaces can reduce electron mobility. The surface morphology of coherently grown β-(In_*x*_Ga_1-*x*_)_2_O_3_ thin films was evaluated using AFM. All samples exhibited high flatness with root-mean-square (RMS) roughness below 0.6 nm (Figure 5). Lower In compositions displayed stripe structures characteristic of homoepitaxial films of (010) β-Ga_2_O_3_. These stripe structures diminished with increasing In composition, and granular features emerged. Several grains were observed at *x* = 0.083. However, these grains, only a few nanometers in height, did not significantly increase the overall RMS roughness. The appearance of grains at *x* = 0.083 may be related to the slight relaxation observed in this composition. Although these small grains could potentially affect electron mobility at heterojunction interfaces, their impact may be minimal, given the overall high flatness of the films observed in our structural characterization. Further electrical characterization of heterojunctions formed with these films is necessary to fully assess their impact on device performance. Despite the minor granular features at higher In compositions, the consistently high flatness across all samples demonstrates the potential for developing β-(In_*x*_Ga_1-*x*_)_2_O_3_-based heterojunction devices. This high-quality surface morphology, combined with coherent growth and controlled composition, provides a promising foundation for future device applications.

**Figure 6.**
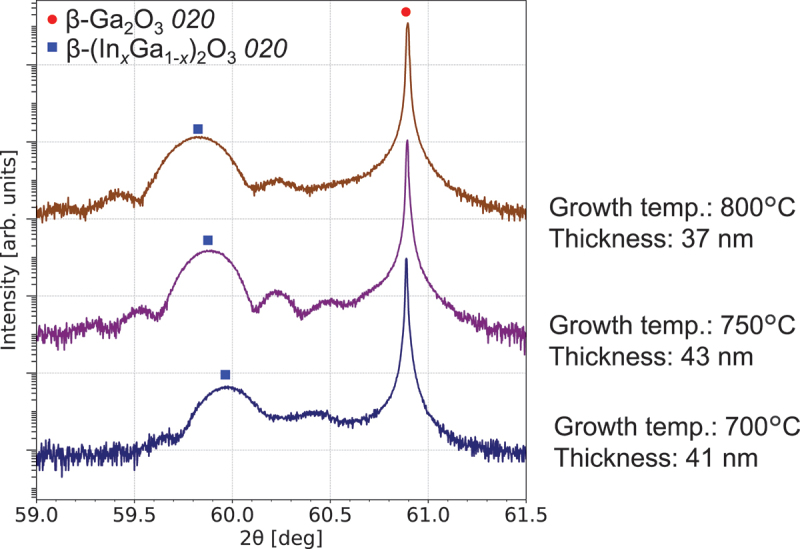
XRD 2θ-ω patterns for β-(In_*x*_Ga_1-*x*_)_2_O_3_ thin films grown at various growth temperatures.

Figure 6 presents the XRD 2θ-ω results for β-(In_*x*_Ga_1-*x*_)_2_O_3_ grown at temperatures of 700°C, 750°C, and 800°C, with an In composition *x* of 0.04 in the solution. The XRD data for 750°C under identical conditions are the same as that shown in Figure 2 for *x* = 0.083, reproduced here for comparison. The diffraction peak positions exhibit a shift towards lower angles as the temperature increases, suggesting reduced In incorporation at lower temperatures.

**Figure 7.**
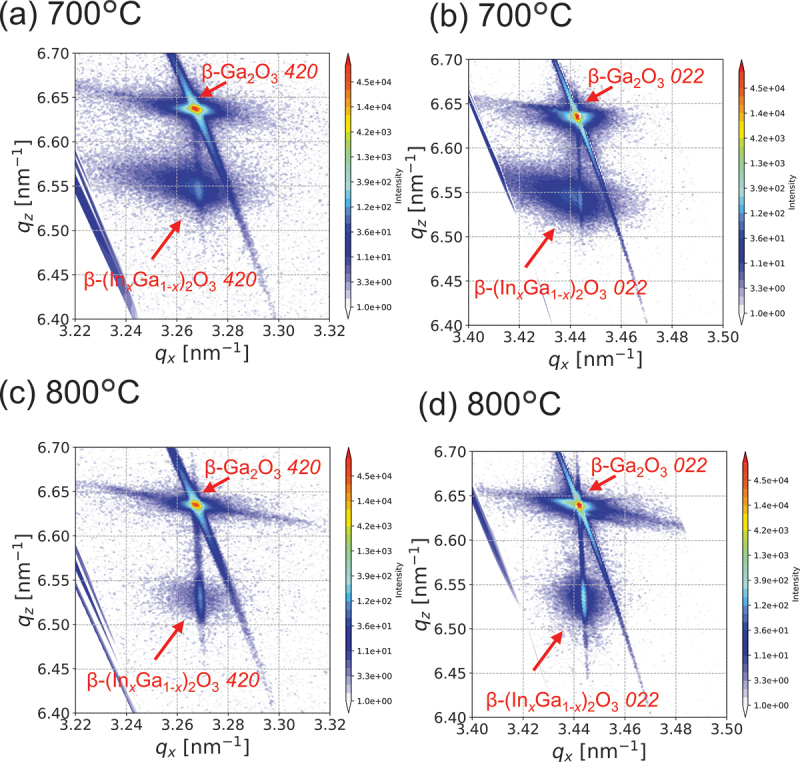
XRD RSM results of β-(In_*x*_Ga_1-*x*_)_2_O_3_ thin films grown at different growth temperatures, focusing on the *420* and *022* reflections.

Figure 7 displays the RSM results for β-(In_*x*_Ga_1-*x*_)_2_O_3_ thin films grown at 700°C and 800°C. The results for 750°C are presented in Figure 3(g,h). The RSM results at 800°C indicate coherent growth. Using the proposed equation, the In composition calculated from the 2θ-ω diffraction peak position for 800°C is *x* = 0.092. In contrast, the 700°C results show clear relaxation. Considering the 2θ-ω results, relaxation begins at lower temperatures despite lower In composition. The difference in In incorporation is attributed to the differing reaction rate constants between GaCl_3_ and InCl_3_. This demonstrates that growth temperature is a crucial parameter for coherent growth of β-(In_*x*_Ga_1-*x*_)_2_O_3_ in mist CVD.

**Figure 8.**
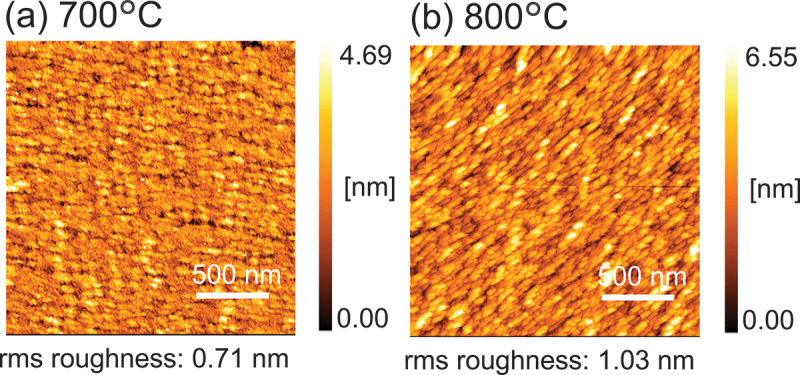
AFM images of β-(In_*x*_Ga_1-*x*_)_2_O_3_ thin films grown at various growth temperatures.

Figure 8 presents AFM results for β-(In_*x*_Ga_1-*x*_)_2_O_3_ thin films grown at 700°C and 800°C. The 750°C result is shown in Figure 5(d). Both 700°C and 800°C samples exhibit greater surface roughness compared to 750°C, indicating that growth temperature also plays a significant role in determining the surface morphology of β-(In_*x*_Ga_1-*x*_)_2_O_3_ in mist CVD.

These findings demonstrate the existence of an optimal growth temperature for β-(In_*x*_Ga_1-*x*_)_2_O_3_ via mist CVD. This result provides crucial guidance for the formation of high-quality β-(In_*x*_Ga_1-*x*_)_2_O_3_ films using mist CVD.

## 4. Conclusions

We successfully grew coherent β-(In_*x*_Ga_1-*x*_)_2_O_3_ thin films on (010) β-Ga_2_O_3_ substrates using mist CVD. A linear relationship between In composition and *b*-axis length was established, facilitating accurate composition determination from XRD peak positions. The films demonstrated high surface flatness, though slight relaxation and granular features appeared at higher In compositions. The findings indicated a critical thickness of approximately 40 nm for *x* = 0.083, significantly lower than theoretical predictions. Future studies should investigate relaxation mechanisms, critical thickness variations, and the electrical characterization of these heterostructures. This work advances ultra-wide bandgap semiconductors and lays the groundwork for novel β-(In_*x*_Ga_1-*x*_)_2_O_3_-based electronic devices.
